# Bone Mass Changes Following Percutaneous Radiofrequency Ablation, Osteoplasty, Reinforcement, and Internal Fixation of Periacetabular Osteolytic Metastases

**DOI:** 10.3390/jcm12144613

**Published:** 2023-07-11

**Authors:** Will Jiang, Dennis L. Caruana, Christopher M. Dussik, Devin Conway, Igor Latich, Julius Chapiro, Dieter M. Lindskog, Gary E. Friedlaender, Francis Y. Lee

**Affiliations:** 1 Department of Orthopaedics & Rehabilitation, Yale School of Medicine, 47 College Street, New Haven, CT 06510, USA; 2 Department of Radiology and Biomedical Imaging, Yale Interventional Oncology, P.O. Box 208042, New Haven, CT 06520, USA

**Keywords:** bone mass, percutaneous, osteolytic lesion, cementoplasty, radiofrequency ablation, periacetabular metastases, periacetabular, internal fixation, ablation

## Abstract

Background: The success of orthopedic interventions for periacetabular osteolytic metastases depends on the progression or regression of cancer-induced bone loss. Purpose: To characterize relative bone mass changes following percutaneous radiofrequency ablation, osteoplasty, cement reinforcement, and internal screw fixation (AORIF). Methods: Of 70 patients who underwent AORIF at a single institution, 21 patients (22 periacetabular sites; average follow-up of 18.5 ± 12.3 months) had high-resolution pelvic bone CT scans, with at least one scan within 3 months following their operation (baseline) and a comparative scan at least 6 months post-operatively. In total, 73 CT scans were measured for bone mass changes using Hounsfield Units (HU). A region of interest was defined for the periacetabular area in the coronal, axial, and sagittal reformation planes for all CT scans. For 6-month and 1-year scans, the coronal and sagittal HU were combined to create a weight-bearing HU (wbHU). Three-dimensional volumetric analysis was performed on the baseline and longest available CT scans. Cohort survival was compared to predicted PathFx 3.0 survival. Results: HU increased from baseline post-operative (1.2 ± 1.1 months) to most recent follow-up (20.2 ± 12.1 months) on coronal (124.0 ± 112.3), axial (140.3 ± 153.0), and sagittal (151.9 ± 162.4), *p* < 0.05. Grayscale volumetric measurements increased by 173.4 ± 166.4 (*p* < 0.05). AORIF median survival was 27.7 months (6.0 months PathFx3.0 predicted; *p* < 0.05). At 12 months, patients with >10% increase in wbHU demonstrated superior median survival of 36.5 months (vs. 26.4 months, *p* < 0.05). Conclusion: Percutaneous stabilization leads to improvements in bone mass and may allow for delays in extensive open reconstruction procedures.

## 1. Introduction

Periacetabular osteolytic lesions present a significant challenge to orthopedic oncological care, given the high weight-bearing requirements of the periacetabular area and severe reductions in mobility that follow. Severe osteolytic destruction in the periacetabular region can threaten the mechanical stability of the pelvic ring. Surgical management has constantly evolved to include open reconstruction and novel percutaneous techniques [1]. Open reconstructions, such as the modified Harrington’s procedure, involve complete resection of the femoral head with endoprosthesis placement along with a cemented acetabular cup, cannulated screw placement, and a reinforcement ring [2]. Open reconstruction has high post-operative complication rates and bleeding risks, especially in cancer patient populations. These procedures rely on systemic agents such as radiation, chemotherapy, and/or bisphosphonates to address local skeletal cancer-induced osteolysis. Percutaneous stabilization offers lower complication rates of 2% compared to 17% seen in open periacetabular reconstruction [3,4]. Percutaneous fixation approaches involving screw fixation, cementoplasty, and ablation have demonstrated strong improvements in functional stabilization and pain improvement, leveling with open approaches in short-term pain and functional improvement [5,6,7,8,9]. Importantly, directly employing percutaneous ablation to the local osteolytic site can provide immediate cancer killing without reliance on systemic agents or radiation—especially in treating periacetabular sites that are already chemo or radio-resistant. Additionally, they provide minimal interruption to life-saving chemotherapy or radiation therapy for other tumor sites, enabling patients to delay open periacetabular reconstruction, including when reconstruction is contraindicated due to comorbidities. While the improvements in early pain and functional outcomes have been well characterized, there is a critical gap in the literature in characterizing bone mass changes following percutaneous stabilization.

Radiofrequency ablation employs thermal-based killing of cancers via percutaneous electrodes set at around 90 °C for 20 min [10]. In oncology, radiofrequency ablation has emerged as a mainstay for both soft tissue tumors and osseous metastases [11]. However, this interventional technique has also gained traction in other uses, such as pain management. Palliative uses of the procedure have been well-characterized in osteoarthritis, joint pain, and neuralgias [12,13]. Radiofrequency ablation has also been used safely in chronic venous insufficiency and atrial fibrillation [14,15]. Returning to the setting of orthopedic care and osteolytic lesions, the use of radiofrequency ablation offers two primary objectives. First, radiofrequency ablation provides immediate cancer killing in the local periacetabular site to stop additional cancer-induced bone loss. Second, radiofrequency ablation demonstrates preliminary evidence of synergy with systemic agents, which may support a patient’s overall cancer care [16]. Osteolytic lesions are a result of severe cancer-induced bone loss and disrupted bone homeostasis [17]. Inversely, if cancer induces bone loss, there is a question as to whether cancer killing will allow for bone mass improvement.

**Figure 1:**
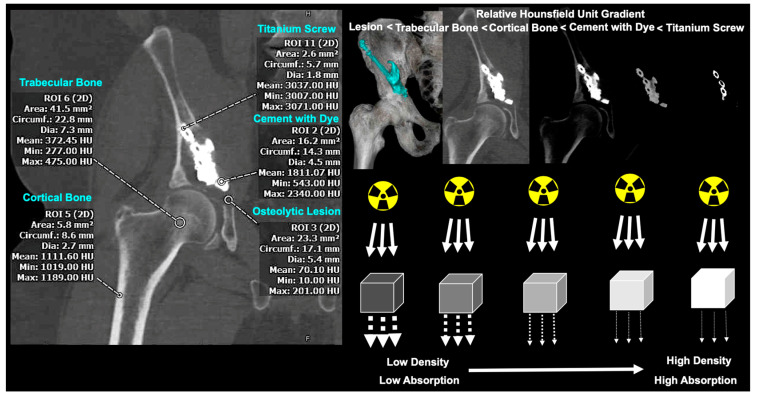
Overview of HU principles underlying CT scan. Titanium screw possesses highest HU values, followed by cement with radio-opaque dye, cortical bone, trabecular bone, and osteolytic lesion site with bony destruction. Higher radiodensity values represent higher absorption of passing radiation beams, which is quantified by increased HU values. HU: Hounsfield Unit.

Computerized tomography (CT) has remained a mainstay in the standard of care for medical and orthopedic management of metastatic skeletal disease. CT scans are interpreted based on Hounsfield Units (HU), which capture radio density based on the attenuation coefficients of radiation beams (Figure 1). While a dimensionless unit, HU ranges are as follows: 100–300 for soft tissue, 300–400 for cancellous bone, 500–1900 for cortical bone, and 2800–3200 for titanium [18]. The emerging paradigm of applying HU in the setting of osteoporosis and fracture risk has been well described in the literature [19]. Moreover, their correlation with the gold-standard dual-energy X-ray absorptiometry (DEXA) scans has ranged from moderate to strong, supporting their sensitivity in capturing bone mass changes [20,21,22]. Because true bone mass cannot be directly measured in a living patient, an indirect assessment via HU changes was performed.

There is a critical need to describe how percutaneous procedures may impact bone mass changes. Moreover, there is a need to determine whether cancer killing by radiofrequency ablation and cement reinforcement with thermal necrosis guide changes in bone mass. This prospective longitudinal cohort study aims to provide one of the first reported measures of indirectly capturing bone mass changes via Hounsfield Unit (HU) assessment following percutaneous radiofrequency ablation, osteoplasty, reinforcement, and internal fixation (AORIF).

## 2. Materials and Methods

### 2.1. Patient Selection

This study represents a prospective longitudinal cohort study that tracked the clinical data and imaging of patients after receiving periacetabular AORIF. To be eligible for inclusion, patients must have obtained a baseline CT scan within the immediate 3 months following their AORIF procedure and a follow-up CT scan at least 6 months post-AORIF. Surgical indications included severe pain, functional limitations in ambulation, and pathological acetabular fracture.

Approval from the university’s Institutional Review Board was obtained prior to study onset. Clinical data were collected from the hospital’s electronic medical record. Outcomes of interest included overall survival (censured at time of data collection), visceral organ progression (either metastatic or primary), skeletal metastases progression, and additional skeletal prophylactic fixation outside of the original surgical site. Progression was defined as either biopsy or radiographical evidence of lesion progression or the development of new lesions following AORIF. Post-operative radiation therapy, chemotherapy, and systemic bisphosphonate administration were also recorded. PathFx 3.0 was utilized to compare expected survival to recorded survival. This machine-learning tool is a clinically validated survival predictor for patients with skeletal metastases [23].

### 2.2. Surgical Technique

**Figure 2:**
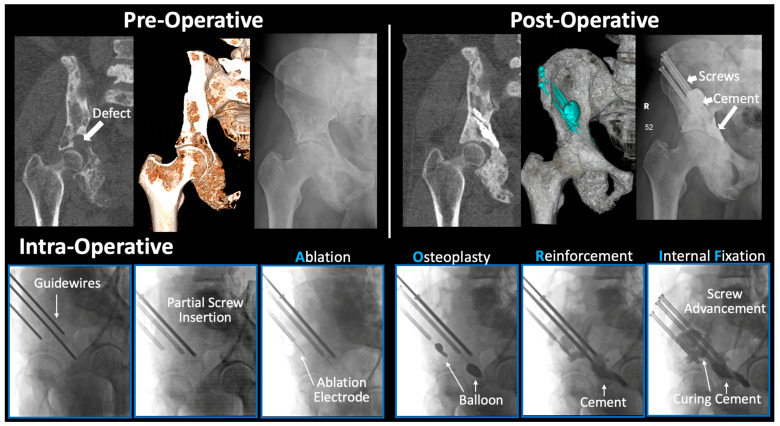
AORIF Procedure Overview. (**Top Panel** (from **left** to **right**)): Radiographical imaging of 62M with metastatic prostate cancer. One week pre-operative (from left to right) imaging demonstrating osteolytic acetabular defect with coronal CT, 3D rendering (internal view), and anterior-posterior pelvis X-ray. Post-operative imaging taken 12-months post-AORIF with PMMA cement and three cannulated screws. (**Bottom Panel** (Intra-Operative)): Initial guidewire placement with partial screw insertion for biopsy. Radiofrequency ablation initiated through insertion via cannulated screws. Balloon inflation creates space and boundaries to facilitate PMMA cement injection. Reinforcement with PMMA cement mixed with Zoledronate provides thermal necrosis of tumor cells and pain relief. Screws are then advanced into their final position in the curing cement for stabilization. AORIF: ablation, osteoplasty, reinforcement, and internal fixation. PMMA: polymethylmethacrylate.

Osteolytic lesion sites were preoperatively localized via CT imaging. Guidewire insertion occurred from either the anterior or posterior iliac crest, avoiding entry through lateral diseased cortical bone (Figure 2). The wires were measured, and 6.5 or 8.0 mm fully threaded titanium cannulated screws (Stryker) were partially inserted along the guidewire. The guidewires were removed, and screwheads were intentionally left external to the skin. This essentially created a percutaneous access route to the periacetabular region. Radiofrequency ablation probes (Medtronic) were advanced through the cannulated screws into areas of concern. Following ablation, balloon osteoplasty was variably performed when warranted via a kyphoplasty balloon (Medtronic). Polymethylmethacrylate (PMMA) cement was then combined with zoledronic acid and mixed (Kyphon Xpede Cement: 69.1% polymethylacrylate, 30% barium sulfate, and 0.9% benzoyl peroxide; mixture was 1 mL zoledronate (4 mg/5 mL) in 20 mL PMMA powder). A cement injector (Medtronic) was inserted through the screws, and cement was slowly injected to fill the lesion cavity [24]. Guidewires were re-inserted, and screws were then advanced into final position within the cement. Guidewires were removed and the wounds closed. All portions of the procedure were performed under fluoroscopic guidance.

### 2.3. Two-Dimensional Assessment of Bone Mass

**Figure 3:**
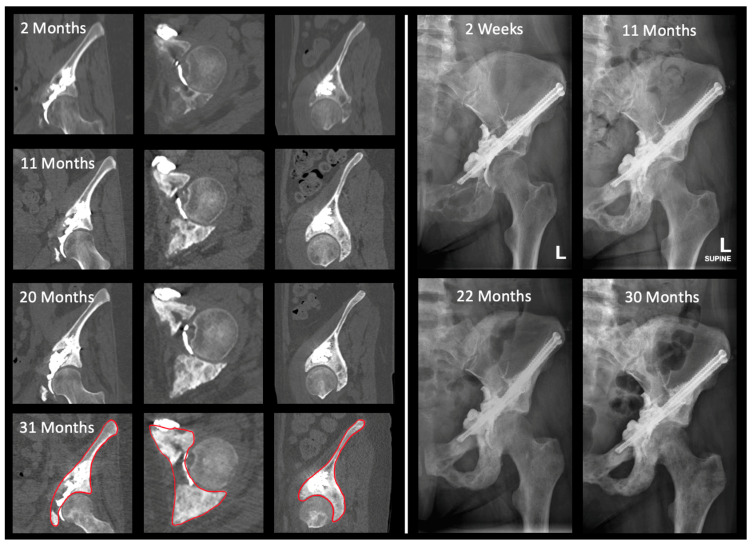
(**Left panel**) Progressive CT imaging showing coronal, axial, and sagittal regions of interest (femur was not included in region of interest selection). Red outline indicates the region of interest used in measurement. (**Right panel**) Progressive plain X-ray radiographs of similar time points. Throughout the post-operative period, the patient exhibited continual visceral organ cancer progression as well as documented skeletal disease progression in the pelvis, femur, sacrum, spine, and humerus. Patient received additional skeletal surgery in the humerus, contralateral acetabulum/femur region, and sacrum. Patient received 30 Gy in 10 fractions of radiation therapy, chemotherapy, and bisphosphonate regiments. Patient is alive and ambulating well at time of study censure. Measured bone mass change between 2 months and 31 months of 35.6% on 3D volumetric grayscale and 2D coronal, axial, and sagittal of 45.9%, 209.5%, and 34.6%, respectively.

No method exists to record absolute bone mass directly. Two-dimensional (2D) analysis was, therefore, an indirect measurement by investigating average HU within a specified region of interest (ROI) on CT scans (Figure 3). Implanted titanium screws were measured as internal controls to ensure CT scanners had detection windows of at least 3071 HU [18]. ROIs were drawn in coronal, axial, and sagittal slices in the bone viewing window on Visage 7.1.17 (Visage Imaging Inc., San Diego, CA, USA). The ROI for periacetabular lesions was defined as the slice that demonstrated maximal diameter of the femoral head. All three reformations were utilized to maximize lesion and bone loss visualization [25]. Only post-operative scans were utilized to account for HU changes from cement and screws. At each time point, identical views and regions of interest were chosen. A 2D analysis was performed on all available CT scans. The average HU within an ROI in each plane was then plotted longitudinally over time. Additionally, a weight-bearing HU score (wbHU) was created by summing the coronal and sagittal HU for a particular scan. These planes were chosen as more clinically relevant for function and stability, given the direction of forces on the acetabulum during ambulation. The wbHU were calculated for scans at 6 months and 1 year (obtained between 9–12 months) and compared to baseline scans.

### 2.4. Three-Dimensional Assessment of Bone Mass

**Figure 4:**
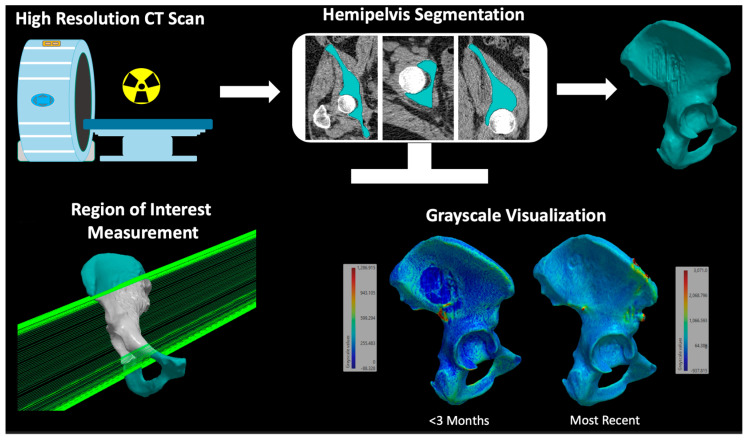
Workflow for the generation of a 3D grayscale-based volumetric assessment of the periacetabular region of interest. A high-resolution CT scan of at least 5.0 mm slice thickness is imported into ScanIP software (2022.12) for segmentation to model the hemipelvis. A region of interest in the periacetabular region is selected for grayscale-based measurement. Hemipelvis based on grayscale values is shown for visualization.

CT scans at baseline and most recent timepoints were exported to Simpleware ScanIP 2022.12 (Synopsys Inc., Mountain View, CA, USA) for three-dimensional (3D) volumetric analysis based on grayscale averages (Figure 4). Auto segmentation was performed for the relevant hemipelvis. Manual slice-by-slice confirmation was conducted to ensure proper modeling of the mask. A 3D periacetabular ROI was selected for grayscale measurement (Figure 4). This region was defined superiorly by the anterior superior iliac spine and posteriorly by transecting the pubis and ischium to capture the full acetabular cup. The grayscale averages of this ROI were compared between baseline and recent CT scans.

## 3. Results

### 3.1. Demographic and Oncologic Treatments

Of the 70 patients who underwent periacetabular AORIF from 2016 to 2022, 32 patients had more than 6 months of survival with follow-up (Table 1). There were 22 hemi-pelvises (21 patients; 13 female and 9 male) included for analysis that met the imaging requirements. The average age was 60.7 (±13.5) years, with an average follow-up of 18.5 (±12.3) months. Most patients received post-operative chemotherapy (18 patients), local radiation (12 patients), or systemic bisphosphonate therapy (15 patients). There were 12 patients (55.0%) who possessed radio- and/or chemo-resistant lesions given a prior history of local radiation and/or systemic chemotherapy before AORIF. A full summary of patient characteristics is provided in Table 1. Fisher’s exact test of radiation therapy, chemotherapy, and bisphosphonate revealed no statistically significant association with bone mass changes.

### 3.2. Two-Dimensional Analysis

**Figure 5:**
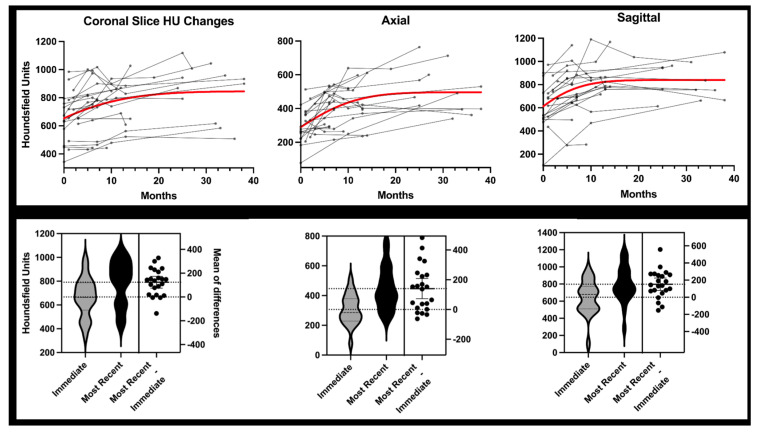
(**Top Row**) A plot of all patient scans collected at various time points following percutaneous ablation, screw fixation, and cementoplasty for periacetabular osteolytic lesions. Red trend line represents cumulative Gaussian distribution for visualization purposes only. (**Bottom Row**) Violin plots of coronal, axial, and sagittal region of interest data. Each patient had a baseline CT scan immediately post-operative (1.2 ± 1.1 months) and most recent post-operative CT scan at time of study censure (20.2 ± 12.1 months). Differences between the immediate baseline scan and most recent scan on each violin plot are the differences in raw Hounsfield units (*p* < 0.05).

CT scans demonstrated an overall trend of increasing HU across all timepoints in all three planes of ROI (Figure 5). From baseline CT (1.2 ± 1.1 months) to most recent CT scan (20.2 ± 12.1 months), the median HU increased 124.0 in coronal, 140.3 in axial, and 151.9 in sagittal plane (all *p* < 0.0005). The wbHU at 6 months and 1 year (12.0 ± 1.6 months) were significantly increased compared to the baseline CT scan, as shown in Table 2.

### 3.3. Three-Dimensional Analysis

The baseline and most recent CT scans were used for volumetric analysis. Grayscale averages demonstrated an increase of 173.4 ± 166.4 from baseline to most recent, representing a magnification of 47.2 ± 55.8%. Two patients demonstrated decreased grayscale values from baseline to the most recent CT measurement; these same two patients also exhibited decreased HU units by 2D analysis.

### 3.4. Survival

**Figure 6:**
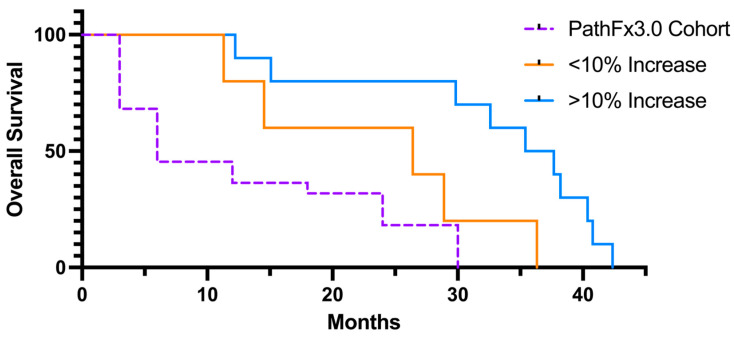
Overall censured survival curve. PathFx3.0 predicted survival of overall cohort included as reference. Patients with at least a 10% increase in relative bone mass as assessed by wbHU demonstrated superior median survival of 36.5 months compared to those who did not (*p* < 0.05). Time point selected was 12 months.

At the time of censor, nine patients (10 sites) had expired from their disease. Skeletal progression or new lesion development was documented in 18 of the 21 patients. Common sites of progression included 9 patients with femur involvement (43%), 11 (52%) patients with pelvis involvement, and 5 (24%) patients with spinal involvement. The expected survival, as calculated by Path Fx 3.0, was 6.0 months, while the observed survival was 27.7 months. At the 1-year CT scan, there was a significant difference in survival among patients who had a >10% increase in wbHU compared to those with a <10% increase (Figure 6). There was no difference between these cohorts with regard to oncologic treatment regimens post-operatively.

## 4. Discussion

**Figure 7:**
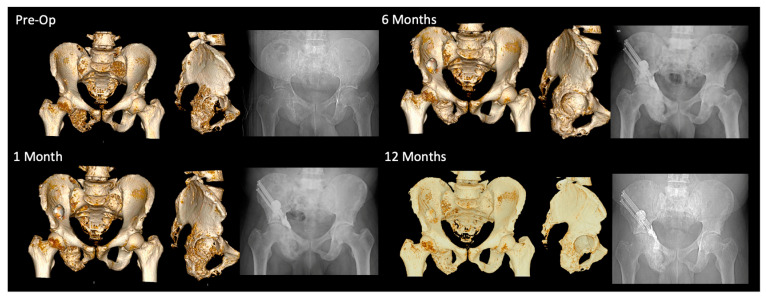
Plain X-ray radiograph and CT reconstruction view of periacetabular disease progression across 12 months. Patient experienced documented visceral organ progression with no history of radiation therapy or chemotherapy. Patient had history of bisphosphonate therapy during the post-operative period and was alive and ambulating well at time of study censure. Measured relative bone mass improvement of 33.7% on 3D volumetric grayscale and 2D coronal, axial, and sagittal of 21.1%, 63.4%, and 16.1%, respectively.

Percutaneous stabilization of periacetabular metastases offers oncologic patients with complex comorbidities an alternative to open fixation, carrying fewer operative complications and quicker recovery times [6,26]. Ablation, cementation, and screw fixation have been characterized in the literature as demonstrating robust restoration of functional status and pain improvement [27]. Percutaneous AORIF is often a same-day procedure with minimal delay to cancer care. Given that delaying the initiation or continuation of life-saving chemotherapies, radiation, and other systemic therapies negatively impacts overall survival, the ability to perform these procedures without interruption of oncologic treatments is critical [28]. Moreover, there is a subset of extremely morbid cancer patients for whom open reconstruction is contraindicated due to severe bleeding and complication risks. Additionally, deploying radiofrequency ablation will provide immediate local cancer control instead of reliance on systemic therapies as with open reconstruction. This is critical as many patients with end-stage cancer demonstrate radio- and/or chemo-resistant osteolytic lesions. While the clinical importance of these minimally invasive interventions is well-documented, there is a paucity of knowledge regarding the local effects on the bone surrounding regions of osteolysis. To our knowledge, this study provides one of the first characterizations of bone mass changes following percutaneous intervention and their efficacy in halting or even reversing cancer-induced bone loss (Figure 7). Although further study is needed, our limited survival data suggest there may be prognostic utility in the surveillance of bone mass changes following prophylactic intervention.

The indirect measurement of bone mass changes via CT HU changes is not a novel approach, even within the setting of cancer care and osteoporosis [19,20,29,30]. Given the frequent usage of CT scans for monitoring in oncologic care, it offers more pragmatic clinical utility as patients are not required to undergo additional scans and radiation exposure risks. Bone possesses a non-homogenous composition of water, organic matrix, and inorganic hydroxyapatite minerals, producing a unique HU signature compared to homogenous materials. CT scanning parameters such as slice thickness, fields of view, and reconstruction protocols may impact exact HU values. However, Free et al. reported that slice thickness and field of view provided negligible changes to HU across different scanners [31]. Even without phantom calibration, modern CT scanners carry maximally reported differences in HU of less than 88 HU [31]. To address inter-scanner variance, titanium screw measurements served as a standardized reference point due to their constant material properties and HU values. Most importantly, identical views and regions of interest were selected to include identical views of the screw and cement to maintain inter-image comparisons. While degradation in cement or titanium is possible, both are highly resistant to corrosion and the bone microenvironment [32,33,34]. Moreover, degradation would only serve to diminish any true difference in HU improvement rather than lead to a falsely elevated improvement. This study only aims to provide longitudinal, relative directionality of bone mass changes over time.

The results of this study demonstrated an increase in the overall density of the periacetabular region following AORIF, as demonstrated by both 2D and 3D analyses. Given the HU constancy of titanium screws and PMMA cement, this is an indirect reflection of bone reconstitution following the procedure. Not only did radiographic density values increase from baseline, but these increases were overall maintained over the course of the study. This demonstrates not only an improvement from baseline but also indicates prevention—or at least a delay—in the resumption of cancer-induced osteolysis in that area. The basis for improvement following AORIF may be for a few identifiable reasons. First, radiofrequency ablation or thermonecrotic effects of the cement may reduce local cancer burden, allowing normal bone homeostasis to be reinstituted around osteolytic sites. Considering a 1 cubic centimeter breast cancer tumor can contain 100 million cells, a reduction from 1 cm to 1 mm reduces the risk of progression from 50% to 0.05% [35]. Stopping cancer-induced bone loss may allow favorability of a more regenerative environment. In murine metastatic models, radiofrequency ablation led to improved bone quality and mineralization profiles [36]. While chemotherapy was not found to be significantly associated with bone mass changes, radiofrequency ablation has limited evidence showing improved chemotherapy sensitization [37,38]. Our PMMA cement included local delivery of zoledronic acid, which may induce bone reconstitution.

Prognostication based on bone mass is of great interest to patients as well as to orthopedic oncologists, medical oncologists, and radiologists, among others. In the entire cohort, survival was found to be extended compared to PathFx3.0 predicted values. Although limited, there is preliminary evidence at 12 months that patients with improvements of at least >10% in wbHU appear to show superior survival. The data are somewhat skewed by the automatic exclusion of patients who passed away within 6 months of their surgery, as they would not meet the imaging requirements for inclusion. However, previous studies without these stringent inclusion criteria similarly demonstrated improved survival when compared to expected values from PathFx 3.0 in patients undergoing acetabular AORIF (15 vs. 3 months) [6]. Given most of these patients were also included in that prior study, the findings of improved survival expectedly correlate with those previous findings. There were no differences in oncologic treatment regimens between these two cohorts. At the 6-month time point, there was a difference noted in survival in a threshold as low as 5%; however, these numbers never reached significance due to a few outliers with early mortality. The AORIF procedure can be conducted in a palliative setting, as well as with the intent of being fully functionally restorative. Patients receiving a palliative intervention may have early mortality regardless of the changes affected in the acetabular region. In its current state, this study is too underpowered to fully explain or correlate the improved survival findings. However, it is a promising finding that for patients who survive for longer periods post-procedure, the changes imparted at the periacetabular region may provide long-term benefits in relation to survival.

Based on indirect assessment, percutaneous interventions of periacetabular lytic lesions promote bone reconstitution and improve bone density, even in the setting of visceral or alternative-site skeletal progression of disease. These changes are seen as soon as 6 months after the procedure and seem to be maintained over time, based on both 2D and 3D analysis. This allows patients to halt or even reverse local cancer-induced bone loss in a high-stress area of the body. There may be some survival benefit for patients who achieve a certain degree of improvement in bone density at 1 year, but further studies are needed to elucidate these prognostic findings.

This study has several limitations. First is the sample size. At this institution, there is no set protocol for CT imaging follow-up after acetabular AORIF. Many patients were lost to follow-up and failed to meet the imaging timepoint requirements of the study. Additionally, metastatic cancer often presents with very limited life expectancy, preventing long-term follow-up. Second, indirect bone mass measurement was employed. Direct measurement of bone mass involves measuring the dry ash weight of hydroxyapatite, which is unrealistic for a clinical study of living patients. HU-based indirect assessments use increased radio density as a systemically and chronologically quantifiable proxy of relative bone mass improvement. This is only an estimate of longitudinal relative bone mass changes [39]. Third, caution must be applied in drawing any conclusions about survival. Given the imaging requirements of at least 6 months post-op, patients with early mortality were not included. However, the minimum requirement for post-operative CT scan is well within the demonstrated average survival from previous studies of the nearly identical patient population [6]. Lastly, no control group was used in the study. A randomized control clinical trial is a difficult task in oncology patients with advanced cancers due to heterogeneity in the cancer itself, varying treatment status, and complex concurrent care.

## 5. Conclusions

Total hip reconstruction in the setting of complex cancer patients poses a high risk of post-operative complications and causes delays in life-saving radiation and systemic therapies. Bone mass underpins functional status and skeletal integrity. Local cancer control via radiofrequency ablation is critical in stopping cancer-induced bone loss and potentially allowing for bone mass improvement. We provide evidence of longitudinal bone mass improvement following minimally invasive ablation and cementoplasty in addition to avoidance of aggravation or prosthesis, as reported in the literature in these patient populations. Initial percutaneous intervention may address not only pain and ambulation but also allow for delays in seeking immediate total reconstruction. This empowers future clinical assessment down the road for open reconstruction depending on cancer progression and life expectancy.

## Figures and Tables

**Table 1:** Summary of patient demographics. Patients were classified having chemotherapy and/or radiation-resistant lesions if the periacetabular osteolytic lesions progressed after initial course of treatment. Progression at other sites outside of the periacetabular region treated by AORIF was also tracked.

Age (u ± SD)	60.7 ± 13.5
Sex	
Male	9 (41%)
Female	13 (59%)
Deceased	10 (45%)
Surgery Side	
Right	8 (36%)
Left	14 (64%)
Primary Malignancy	
Breast	8 (36%)
Lung	4 (18%)
Multiple Myeloma	4 (18%)
Other	6 (27%)
Bisphosphonates	15 (68%)
Chemotherapy	18 (82%)
Radiation Therapy	12 (55%)
Progression	
Visceral	20 (91%)
Skeletal	18 (82%)

**Table 2:** Top Panel: Comparison between the baseline (immediate post-op) CT scan and the scan at the longest-available follow-up with difference and *p*-values based on paired T-test. Data shown for 2D (coronal, axial, sagittal) and 3D grayscale-based assessment of region of interest. Bottom Panel: Analysis of wbHU (sum of coronal and sagittal) at 6 months and 12 months. wbHU: weight-bearing Hounsfield unit.

	**Baseline (u)**	**Longest Follow-Up (u)**	**Difference**	***p*-Value**
Coronal	666.9 ± 164.3	790.9 ± 189.0	124.0 ± 112.3	<0.0001
Axial	306.3 ± 102.1	446.6 ± 143.6	140.3 ± 153.0	0.0003
Sagittal	645.6 ± 197.0	797.5 ± 207.6	151.9 ± 162.4	0.0003
3D Grayscale	472.0 ± 128.1	645.5 ± 128.1	173.4 ± 166.4	<0.0001
**wbHU**				
	**Baseline**	**Comparison**	**Difference**	***p*-Value**
6 Months Comparison (*n* = 20)	1336.7 ± 271.9	1502.0 ± 351.1	165.3 ± 164.0	0.0002
12 Months Comparison (*n* = 15)	1338.2 ± 346.9	1569.1 ± 279.2	230.8 ± 214.0	0.0009

## Data Availability

The data are not publicly available due to patient privacy. The data presented in this study are available on request from the corresponding author although restrictions may apply to protect patient data.
